# Preschoolers’ exclusion detection and empathy: the mediating role of moral evaluation

**DOI:** 10.3389/fpsyg.2026.1805274

**Published:** 2026-07-21

**Authors:** Xuerong Zhao, Tianyu Zhang, Yaxin Yan, Xiangkui Zhang

**Affiliations:** 1 School of Psychology, Northeast Normal University, Changchun, China; 2 College of Applied Technology, Anshan Normal University, Anshan, China

**Keywords:** empathy, exclusion detection, moral evaluation, preschool children, social exclusion

## Abstract

**Introduction:**

Empathy encompasses emotional, cognitive, and behavioral components. Although empathy for physical pain in preschoolers is well documented, less is known about their responses to others’ social pain. Whether preschoolers empathize with socially excluded individuals and how exclusion detection and moral evaluation affect that empathy remain unclear. Our study examined the development of preschoolers’ exclusion detection, moral evaluation, and empathy, and how these aspects relate to one another.

**Methods:**

We conducted a cross-sectional study with 98 preschool children aged 3 to 5 years in northeastern China. Using the Cyberball paradigm, children observed a social exclusion scenario and answered questions to assess exclusion detection, moral evaluation, and empathy. We also investigated whether moral evaluation mediated the relationship between exclusion detection and empathy.

**Results:**

Exclusion detection, moral evaluation, and cognitive empathy develop rapidly with age, with 5-year-olds outperforming 3-year-olds in these abilities. Additionally, children’s moral evaluation of the excluder mediated the relationship between exclusion detection and emotional and cognitive empathy, accounting for 19.6% and 19.7% of the total effect, respectively. Similarly, the moral evaluation of exclusionary behavior mediated the relationship between exclusion detection and emotional, cognitive, and behavioral empathy, accounting for 21.4%, 32.4%, and 24% of the total effect, respectively.

**Discussion:**

These findings suggest that children who excel at detecting exclusion and making suitable moral evaluations tend to display higher levels of empathy.

## 1 Introduction

Social exclusion is a common form of social distress in interpersonal interactions. It can negatively impact children by lowering self-esteem, increasing anxiety and depression, and causing academic struggles (Wirth et al., 2020). Researchers consider exclusion a form of relational aggression or bullying, and argue that increased empathy can prevent and mitigate such behaviors (Vlachou et al., 2011). One study of adolescents found that those with higher empathy levels were more likely to empathize with and protect victims. In comparison, those with lower empathy levels were more likely to reinforce bullying behaviors and exhibit bystander indifference (Hikmat et al., 2024). Empathy is often described as the ability to understand and share others’ feelings and respond to them appropriately. It includes emotional, cognitive, and behavioral aspects. Emotional empathy involves sharing and experiencing another person’s feelings, while cognitive empathy focuses on recognizing and understanding emotions in oneself and others. Behavioral empathy refers to the willingness, motivation, or actions to help others (Guo and Wu, 2021). Although many studies have demonstrated that empathy can alleviate the adverse effects of physical pain and social distress, most of this research has focused on adolescents and adults (Ferguson et al., 2024; Goldstein et al., 2016). Research on young children’s empathy for pain has primarily focused on physical pain (Yan et al., 2021). Whether children can empathize with social pain, such as exclusion, is a matter of controversy.

Exclusion and bullying are rare in early childhood (Douvlos, 2019). However, from an evolutionary psychological perspective, humans are vulnerable to exclusion from infancy and show a basic understanding of it (Quadrelli et al., 2023). Research has demonstrated that infants aged 7–9 months, when observing a character excluded from group interactions, expect neutral characters to approach and include the excluded individual in later interactions (Prendergast, 2019). The ability to understand and detect exclusion grows quickly during early childhood. Two-year-olds randomly choose either the excluder or the excluded person as a playmate, but three-year-olds are more likely to pick the excluded individual (Hwang et al., 2017). Additional studies have shown that most children develop verbal exclusion detection abilities by ages 5 to 6. Although 3- to 4-year-olds cannot verbally state exclusion, they show an implicit understanding through their choice of partners and behavior (Hwang and Markson, 2020; Woodward et al., 2023).

Older preschoolers not only detect social exclusion but also make moral evaluations. Research indicates that 3- to 4-year-olds have limited ability to detect exclusion and often hold relatively positive evaluations of the excluder. In contrast, 5- to 6-year-olds not only detect exclusionary behavior but are also more likely to describe the excluder as “mean” (Hwang and Markson, 2020). Another study also found that two-thirds of 4- to 6-year-olds could recall the identity of the excluder, which gave them more negative evaluations (Woodward et al., 2023). In summary, children with detection abilities are more likely to perceive the excluder as “mean,” suggesting that bottom-up “exclusion detection” underpins top-down “moral evaluation” (Heyes, 2018). However, it is important to note that this study did not assess children’s empathy toward the excluded individual.

Whether children can empathy toward the excluded individual remains a topic of debate. Children aged 4–5 can recognize that the excluded person is sad. However, they do not feel sad for the excluded (Song et al., 2015), indicating that children show cognitive empathy toward the excluded but lack emotional empathy. Another study involving children aged 3–7 reached similar conclusions (Stengelin et al., 2022). However, two additional studies with children aged 5–12 and 8–11 found that children can show emotional empathy toward the excluded, with differences observed between in-group and out-group emotional empathy (Jones and Rutland, 2020; Masten et al., 2010). Overall, the different findings among these studies result from variations in the social exclusion paradigms used and the specific dimensions of empathy measured. Additionally, the use of geometric shapes as experimental materials in some studies may influence results due to their limited ecological validity (Song et al., 2015; Stengelin et al., 2022). As a result, previous studies have not yet provided definitive explanations regarding the development characteristics of emotional, cognitive, and behavioral empathy in preschool children.

The theory of evaluative empathy and the third-person evaluative framework for children’s emotional understanding suggest that empathy depends on two main cognitive processes, which are noticing emotional stimuli or events and making cognitive evaluations (Doan et al., 2025; Wondra and Ellsworth, 2015). Research with children aged 4–8 years showed higher levels of emotional empathy and empathic concern toward moral dolls displaying physical pain compared to immoral dolls (Wang, 2023). Another study also found that children aged 6–7 were more likely to include peers from different ethnic backgrounds than aggressive peers. Children viewed aggressive peers as amoral, lacking empathy and altruism (Walker et al., 2022). We can infer that when children observe social exclusion in interactions and evaluate it as immoral, they may develop empathy for the excluded individual. However, current research has not thoroughly examined the connections among detecting exclusion, moral evaluation, and different aspects of empathy, which limits our understanding of children’s cognitive and emotional processes when they witness social exclusion.

To address gaps in previous research, we used a Cyberball paradigm to examine how children aged 3 to 5 years develop exclusion detection, moral evaluation, and empathy, as well as the relationships among these abilities. This paradigm has been shown to have good ecological validity for preschool-aged children (Hwang and Markson, 2020; Marinović et al., 2017). We hypothesized that children’s abilities in exclusion detection, moral evaluation, and empathy would improve with age. We also suggested that exclusion detection would affect children’s empathy toward excluded individuals through moral evaluation. Specifically, children’s exclusion detection would shape their moral evaluations of the excluder’s character and behavior, which then affects their emotional and cognitive empathy toward the excluded child. When children detect exclusion and evaluate the excluder and their behavior as immoral, they may show greater emotional and cognitive empathy toward the excluded individual.

## 2 Materials and methods

### 2.1 Participants

A total of 100 children from northeastern China participated in the study. The final sample consisted of 98 children (*M*_*age*_ = 54.72 months, *SD* = 9.76), including 46 boys and 52 girls. We included children with adequate language skills to understand and verbally respond to questions. Two children were excluded due to their inability to comprehend or answer verbally. All participants were typically developing native Chinese speakers. The final sample comprised 27 three-year-olds, 36 four-year-olds, and 35 five-year-olds.

### 2.2 Material

#### 2.2.1 Exclusion detection task

We used the classic Cyberball paradigm (Williams and Jarvis, 2006), which has been used with children as young as 1 year old (Quadrelli et al., 2023). We presented inclusion and exclusion conditions of the game to preschool children on a 14-inch computer screen. Each game involved three players and 30 ball passes. In the inclusion game, each player received 10 passes. In the exclusion game, the first six passes were evenly distributed among the three players, so that each player received the ball twice; the remaining 24 passes were made only between the two green-shirted players, thereby excluding the blue-shirted player. After the exclusion condition, children answered a brief question (e.g., “What happened?” or “How did the three players pass the ball?”) to assess their ability to detect exclusion. Responses were coded as 1 when children explicitly mentioned exclusion-related information, such as “The two green-shirted players did not pass the ball to the blue-shirted player” or “The two green-shirted players stopped playing with the blue-shirted player after initially playing together.” Responses were coded as 0 when children did not mention exclusion-related information, such as “I don’t know what happened,” “The three players played together,” or “They received the ball equally” (Song et al., 2015; Woodward et al., 2022). All children completed the subsequent moral evaluation and empathy assessment tasks, regardless of their exclusion detection score. This procedure allowed us to examine the associations among exclusion detection, moral evaluation, and empathy.

#### 2.2.2 Moral evaluation task

Children’s moral evaluations of exclusion were assessed by asking children to evaluate both the excluders and their exclusionary behavior. Consistent with previous research on 4- to 8-year-old children (Li et al., 2019; Wang, 2023), we used a 5-point pictorial Likert-type scale with two questions: (1) “Are the two green-shirted players nice or mean? How much?” (assessing evaluations of the excluders) and (2) “Are the actions of these two green-shirted players nice or mean? How much?” (assessing evaluations of exclusionary behavior). The order of the two questions was counterbalanced across participants: half of the children answered the excluder-evaluation question first, followed by the behavior-evaluation question, whereas the other half answered the questions in the reverse order. Children’s responses were scored from −2 to 2: very mean (−2), somewhat mean (−1), neutral, unsure, or do not know (0), somewhat nice (1), and very nice (2).

Before viewing the social inclusion and exclusion games, the experimenter introduced the 5-point pictorial scale to ensure that children understood the response options. Specifically, children were told that the thumbs-down icons represented “mean,” the thumbs-up icons represented “nice,” and the circle represented “neutral,” “unsure,” or “don’t know.” They were also told that the sizes of the thumbs-down and thumbs-up icons indicated the degree of meanness or niceness. Children were then asked to identify which picture represented “mean,” which represented “nice,” and which represented “neutral/unsure/don’t know.” If a child answered incorrectly, the experimenter repeated the explanation and administered the comprehension check again. Children who did not demonstrate understanding of the scale after three explanations and comprehension checks were considered unable to comprehend the experimental materials and therefore did not proceed to the subsequent tasks.

#### 2.2.3 Empathy assessment task

We assessed children’s emotional, cognitive, and behavioral empathy (Stengelin et al., 2022). Consistent with previous studies assessing empathy in preschool children, emotional empathy was evaluated using a 5-point pictorial emotion scale and a single-item measure (Guo and Wu, 2021). Before viewing the social inclusion and exclusion games, the experimenter introduced the 5-point emotion scale and confirmed that children understood the meanings of the facial expressions. Children were asked, “How do you feel after watching the second game?” To avoid influencing children’s empathy-related responses, the experimenter did not introduce the word “exclusion” during the empathy assessment. If children spontaneously mentioned exclusion-related information in the exclusion detection task, the experimenter did not provide additional labels for, or explanations of, the event before asking the empathy questions. Responses were coded according to whether children’s emotional valence matched the presumed sadness of the blue-shirted player. Specifically, responses were coded according to whether children’s emotional valence was congruent with the presumed emotional state of the excluded player. Responses indicating sadness were coded as emotional congruence, or emotional matching, and were interpreted as emotional empathy; neutral responses were coded as no emotional empathy; and responses indicating happiness were coded as counter-empathy (Hudson et al., 2019; Smith-Flores et al., 2023). The raw emotion scores ranged from −2 to 2, with −2 indicating very sad, −1 indicating somewhat sad, 0 indicating neutral, unsure, or did not know, 1 indicating somewhat happy, and 2 indicating very happy. For analyses, these scores were reverse-coded so that higher scores indicated stronger emotional empathy.

Cognitive empathy was assessed as children’s ability to recognize and understand the excluded player’s emotion and to reflect on children’s own emotional experience. It was assessed with three items: (1) “What emotion is the blue-shirted player feeling?”; (2) “Why is the blue-shirted player feeling this emotion?”; and (3) “Why are you feeling this emotion?” For the first item, children responded using the same 5-point pictorial emotion scale used for emotional empathy, and scores were reverse-coded so that higher scores indicated more accurate recognition of the excluded player’s sadness. Children’s responses to the two explanation questions were scored as 0 (irrelevant or did not know), 1 (vaguely related to exclusion), or 2 (accurately attributing the blue-shirted player’s sadness or the child’s own emotional response to the exclusion event). The cognitive empathy score was calculated as the sum of the three items after recoding, with higher scores indicating greater cognitive empathy, that is, more accurate recognition and understanding of the excluded player’s emotion and clearer differentiation between the child’s own emotional experience and the excluded child’s emotional state.

We assessed behavioral empathy with a single question: “If you were next to the blue-shirted player, what would you do?” Responses were scored according to whether children indicated willingness to help (0 = no help; 1 = help). The empathy assessment included five questions. If a child was unable to answer three or more questions, the experimenter rephrased and repeated the questions. If the child still could not answer three or more questions, the child was considered unable to understand the experimental materials or respond orally to the questions and therefore did not proceed to the subsequent experimental tasks.

### 2.3 Procedure

**FIGURE 1:**
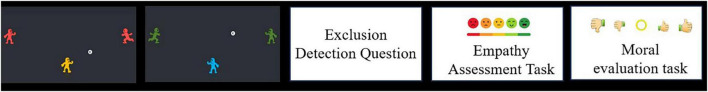
Schematic illustration of the experimental procedure.

All children followed a consistent protocol: They viewed the inclusion and exclusion games and then completed the exclusion detection, empathy, and moral evaluation tasks. This study was not preregistered. Before the games, the experimenter introduced the players and explained the 5-point pictorial Likert-type scales for emotion and moral evaluation to ensure that children understood the materials, response options, and procedure. Written informed consent was obtained from children’s parents or legal guardians before participation, and children provided verbal assent before the assessment began. The study was approved by the Ethics Committee of the School of Psychology at the first author’s university and was conducted with permission and support from the local educational authorities, kindergarten directors, and teachers. Children were assessed individually in a designated quiet room at their kindergarten. Figure 1 illustrates the procedure.

### 2.4 Data analysis

Exclusion detection and behavioral empathy were coded as dichotomous variables, while moral evaluations were treated as an ordinal variable with three levels (immoral, moral, and neutral/unsure). Emotional and cognitive empathy were measured as continuous variables. Consequently, we employed various data analysis methods to account for age-related differences in these abilities. First, chi-square tests were used to assess the impact of age on exclusion detection and behavioral empathy. Second, Kruskal-Wallis tests were used to examine the influence of age on moral evaluation. Finally, a one-way ANOVA was used to analyze the effect of age on emotional and cognitive empathy.

All data analyses were conducted using R software, version 4.2.0 (R Core Team, 2022). We initially calculated descriptive statistics for exclusion detection, moral evaluation, and empathy tasks, then tested the effects of age on these three abilities, examining their correlations. Finally, using the mediation package (Imai et al., 2010), we explored whether moral evaluation mediated the relationship between detection and empathy.

## 3 Results

### 3.1 Effects of age on exclusion detection, moral evaluation, and empathy

Table 1 presents the specific results for age-related differences in the three abilities. A chi-square test of independence indicated a significant effect of age on exclusion detection, χ^2^ (2) = 7.18, *p* = 0.028, Cramér’s *V* = 0.27. A homogeneity-corrected Fisher’s exact test showed that 5-year-olds detected exclusion more accurately than 3-year-olds (*p* = 0.033), with no significant differences between 3- and 4-year-olds (*p* = 0.233) or between 4- and 5-year-olds (*p* = 0.372). Because 55% of 3-year-olds detected exclusion whereas 45% did not, we further compared these two groups on moral evaluation and empathy. There were no significant differences between 3-year-olds who detected exclusion and those who did not in their moral evaluations of the excluders, χ^2^ (1) = 1.20, *p* = 0.239, Cramér’s *V* = 0.29; moral evaluations of the exclusionary behavior, χ^2^ (2) = 4.81, *p* = 0.078, Cramér’s *V* = 0.42; or behavioral empathy, χ^2^ (1) = 0.64, *p* = 0.398, Cramér’s *V* = 0.24. Because of the small group sizes and unequal standard deviations, Welch’s independent-samples *t* tests were used to compare emotional and cognitive empathy. Three-year-olds who detected exclusion showed higher emotional empathy, *t* (22.08) = -3.20, *p* = 0.004, Cohen’s *d* = 1.16, and higher cognitive empathy, *t* (24.90) = −2.88, *p* = 0.008, Cohen’s *d* = 1.09, than those who did not detect exclusion.

**TABLE 1:** Descriptive statistics and results of age differences in various abilities among preschool children.

Variable	Age	*n*	%	*M* ± *SD*	*χ^2^*	*p*	*F*	*df*	*Cramér’s V*	*η^2^*
Exclusion detection	3	15	55.6		7.18	0.028			0.27	
	4	27	75.0							
	5	30	85.7							
Moral evaluation of behavior	3	15	55.6		5.87	0.053				0.04
	4	29	80.6							
	5	31	88.6							
Moral evaluation of the excluder	3	11	40.7		11.86	0.003				0.10
	4	24	66.7							
	5	29	82.9							
Behavioral empathy	3	19	70.4		4.55	0.103			0.22	
	4	29	80.6							
	5	32	91.4							
Emotional empathy	3			−0.44 (1.28)		0.159	1.88	2, 95		0.04
	4			−0.03 (1.50)						
	5			0.23 (1.26)						
Cognitive empathy	3			2.19 (2.66)		0.018	4.17	2, 95		0.08
	4			3.58 (2.72)						
	5			4.06 (2.40)						

Kruskal–Wallis tests indicated that age significantly influenced children’s moral evaluations of excluders, χ^2^ (2) = 11.86, *p* = 0.003, η^2^ = 0.10. Dunn’s pairwise comparisons revealed that 4- and 5-year-olds more frequently considered excluders immoral compared to 3-year-olds (*p* = 0.0003 and *p* = 0.017). There was no significant difference between 4- and 5-year-olds (*p* = 0.077). Regarding moral evaluations of exclusionary behavior, the age effect was marginally significant, χ^2^ (2) = 5.87, *p* = 0.053, η^2^ = 0.04. Significantly more 5-year-old (*p* = 0.008) and 4-year-old (*p* = 0.047) children reported exclusionary behaviors as immoral compared to 3-year-old children, with no significant difference between 4- and 5-year-olds (*p* = 0.217).

The ANOVA revealed significant age differences in cognitive empathy, *F* (2, 95) = 4.17, *p* = 0.018, η^2^ = 0.04. Follow-up tests indicated that 5-year-olds performed better than 3-year-olds, *t* (60) = 2.85, *p* = 0.018; however, there were no significant differences between the 3- and 4-year-olds or between the 4- and 5-year-olds. Age effects were not significant for emotional empathy, *F* (2, 95) = 1.88, *p* = 0.159, η^2^ = 0.08, and for behavioral empathy, χ^2^ (2) = 4.55, *p* = 0.103, Cramér’s *V* = 0.22.

### 3.2 Correlation analysis of exclusion detection, moral evaluation, and empathy

Bivariate correlations showed moderate positive relationships between exclusion detection and both emotional empathy [*r* = 0.44, *t* (96) = 4.73, *p* < 0.001] and cognitive empathy [*r* = 0.48, *t* (96) = 5.39, *p* < 0.001]. The link with behavioral empathy was moderate to strong (Cramér’s *V* = 0.39). These results suggest that children with better exclusion detection skills are more likely to demonstrate greater empathy toward excluded peers. Additionally, exclusion detection was moderately associated with the moral evaluation of the excluder (Cramér’s *V* = 0.31) and exclusionary behavior (Cramér’s *V* = 0.29).

Children’s moral evaluations of the excluder and of exclusionary behavior were significantly associated with empathy. The moral evaluations of the excluder and exclusionary behavior accounted for 10% and 15% of the variance in emotional empathy (η^2^ = 0.10, η^2^ = 0.15), and 17% and 35% in cognitive empathy (η^2^ = 0.17, η^2^ = 0.35), respectively. Associations with behavioral empathy were weak to moderate (Cramér’s *V* = 0.21 for excluder evaluations; Cramér’s *V* = 0.31 for behavior evaluations).

Children’s moral evaluations of the excluder and exclusionary behavior were strongly correlated, Cramér’s *V* = 0.66. McNemar’s test was conducted to examine whether there were significant differences in the number of children who evaluated the excluder and the exclusionary behavior as immoral. A higher number of children evaluated the exclusionary behavior as immoral (75 out of 98, 77%) compared to those who evaluated the excluder as immoral (64 out of 98, 65%), χ^2^ (1) = 6.67, *p* = 0.01, Φ = 0.65. This indicates that children may distinguish between evaluating an individual’s behavior and evaluating the individual as immoral. This distinction seems more evident in younger children.

### 3.3 Analysis of how moral evaluation mediates the relationship between exclusion detection and empathy

We examined whether children’s moral evaluations mediated the relationship between exclusion detection and empathy, as shown in Table 2. Using nonparametric bootstrapping with 5,000 resamples to estimate the average causal mediation effect (ACME), average direct effect (ADE), total effect, and proportion mediated, the evaluations of both the excluder and the exclusionary behavior partially mediated the link with emotional empathy, accounting for 19.6% and 21.4% of the total effect, respectively, and with cognitive empathy, accounting for 19.7% and 32.4%, respectively. For behavioral empathy, only evaluations of exclusionary behavior showed partial mediation, accounting for 24%.

**TABLE 2:** Mediation analysis of the effect of exclusion detection on empathy through moral evaluation.

Mediating variable	Outcome variable	Effect	*b*	95% *CI*	*p*
Evaluations of excluders	Emotional empathy	ACME	0.264	(0.048,0.610)	0.01
		ADE	1.080	(0.500, 1.590)	< 0.001
		Total effect	1.344	(0.852, 1.800)	< 0.001
		Prop. Mediated	0.196	(0.036, 0.500)	0.01
Evaluations of behavior	Emotional empathy	ACME	0.288	(0.049, 0.640)	0.008
		ADE	1.057	(0.455, 1.620)	0.004
		Total Effect	1.344	(0.836, 1.820)	< 0.001
		Prop. Mediated	0.214	(0.033, 0.530)	0.008
Evaluations of excluders	Cognitive empathy	ACME	0.573	(0.135, 1.220)	0.005
		ADE	2.335	(1.762, 3.460)	< 0.001
		Total Effect	2.908	(1.837, 3.990)	< 0.001
		Prop. Mediated	0.197	(0.047, 0.440)	0.005
Evaluations of behavior	Cognitive empathy	ACME	0.941	(0.301, 1.740)	0.002
		ADE	1.967	(1.010, 2.910)	< 0.001
		Total Effect	2.908	(1.809, 3.960)	< 0.001
		Prop. Mediated	0.324	(0.121, 0.560)	0.002
Evaluations of excluders	Behavioral empathy	ACME	0.0387	(–0.010, 0.110)	0.135
		ADE	0.235	(0.036, 0.440)	0.019
		Total Effect	0.274	(0.064, 0.480)	0.008
		Prop. Mediated	0.142	(−0.047, 0.560)	0.139
Evaluations of behavior	Behavioral empathy	ACME	0.066	(0.0003, 0.170)	0.047
		ADE	0.208	(0.015, 0.410)	0.034
		Total Effect	0.274	(0.072, 0.480)	0.009
		Prop. Mediated	0.240	(−0.003, 0.780)	0.054

## 4 Discussion

The present study examined how preschool children detect social exclusion, morally evaluate both exclusionary behavior and the excluders, and respond empathically to an excluded peer. The findings indicated developmental differences in exclusion detection, moral evaluation, and empathy. Specifically, 5-year-olds showed better exclusion detection and cognitive empathy than 3-year-olds, whereas 4- and 5-year-olds were more likely than 3-year-olds to evaluate both the excluders and the exclusionary behavior as mean. In addition, 3-year-olds who detected exclusion showed higher emotional and cognitive empathy than 3-year-olds who did not detect exclusion. Children also distinguished between evaluations of exclusionary behavior and evaluations of the excluders. More children judged the behavior as immoral than judged the excluders themselves as immoral, and this distinction was especially evident among younger children. Mediation analyses further showed that moral evaluation partially mediated the association between exclusion detection and empathy. In particular, moral evaluation of exclusionary behavior mediated the relation between exclusion detection and emotional, cognitive, and behavioral empathy, whereas moral evaluation of the excluder mediated only emotional and cognitive empathy. Taken together, these findings suggest that preschoolers’ empathy toward an excluded peer depends not only on whether they notice that exclusion has occurred but also on whether they appraise the exclusion as morally wrong. These findings extend previous work on preschoolers’ responses to social exclusion by showing that moral evaluation, particularly evaluation of exclusionary behavior, helps explain why children who detect exclusion are more likely to show emotional, cognitive, and behavioral empathy toward an excluded peer.

The age-related improvement in exclusion detection is consistent with previous research showing that preschoolers’ understanding of exclusion develops rapidly across early childhood. Although some 3- to 4-year-olds can show implicit sensitivity to exclusion, older preschoolers are better able to verbally identify the excluded peer and explain the exclusion event (Hwang and Markson, 2020; Woodward et al., 2023). In the present study, 5-year-olds detected exclusion more accurately than 3-year-olds, suggesting that explicit representations of exclusion in peer interactions become more stable during the preschool years. The present study found 3-year-olds further qualified this pattern: More than half detected exclusion, whereas a substantial minority did not. Those who detected exclusion showed higher emotional and cognitive empathy than those who did not; however, the groups did not differ significantly in moral evaluation or behavioral empathy. This finding suggests that explicit detection may help young children identify the excluded peer, share the peer’s distress, and explain the peer’s sadness. Thus, exclusion detection may support, rather than automatically produce, emotional and cognitive empathy in response to social pain (Doan et al., 2025; Wondra and Ellsworth, 2015). In contrast, its associations with moral evaluation and behavioral empathy appear less direct. This dissociation is consistent with evidence that recognizing exclusion does not necessarily translate into more negative evaluations of excluders, stronger preferences for includers, or later inclusion behavior among younger preschoolers (Stengelin et al., 2022; Woodward et al., 2022).

Children’s moral evaluation of social exclusion increased with age. More 4- and 5-year-olds than 3-year-olds judged both the excluders and the exclusionary behavior as immoral. This pattern is consistent with developmental evidence suggesting that, with age, children increasingly consider welfare, fairness, intention, and responsibility when making moral evaluations of social exclusion (Jambon and Smetana, 2018; Seucan et al., 2024; Smetana and Ball, 2018). The present study further showed that preschoolers distinguished evaluations of exclusionary behavior from evaluations of the excluders. Specifically, more children judged the exclusionary behavior as immoral than judged the excluders themselves as immoral, and this distinction was particularly pronounced among younger children. One explanation is that behavior-based moral evaluation may emerge earlier than actor-based moral evaluation (Dahl and Killen, 2018; Margoni and Surian, 2016). For 3- to 4-year-olds, the exclusionary behavior was concrete and perceptually salient, as two players stopped passing the ball to the blue-shirted player. Younger children may therefore have judged the behavior as wrong because it violated fairness and caused social harm (Dahl and Killen, 2018; Smetana and Ball, 2018). In contrast, judging the excluders as immoral required integrating the actors’ identity, intentionality, responsibility, and stable moral character (Cushman et al., 2013; Gill et al., 2023; Killen et al., 2011; Margoni and Surian, 2016). Because these abilities continue to develop during the preschool years, younger children may condemn the exclusionary behavior without consistently extending this judgment to the excluders themselves (Killen et al., 2011; Vaish et al., 2010). By age 5, children’s more developed exclusion detection, memory for actors, intention understanding, and executive functioning may support more consistent evaluations of both the behavior and the excluders (Hwang and Markson, 2020; Woodward et al., 2023). Thus, these findings suggest a developmental shift from concrete behavior-based moral evaluation to more integrated actor-based moral evaluation during the preschool years (Cushman et al., 2013; Hwang and Markson, 2020; Margoni and Surian, 2016; Woodward et al., 2023).

The present study found that the clearest age-related difference emerged for cognitive empathy, with 5-year-olds performing better than 3-year-olds. Emotional empathy and behavioral empathy showed weaker or non-significant age-related patterns and therefore should be interpreted more cautiously. This pattern suggests that preschoolers’ empathy toward an excluded peer in a social-exclusion context develops most clearly in their ability to understand the excluded child’s emotion and the reasons for that emotion. In the present study, cognitive empathy required children to identify the excluded child’s emotion, attribute it to the exclusion event, and provide a reason for that emotional state. These abilities are cognitively demanding because they rely on language, emotion understanding, theory of mind, and self–other differentiation (Decety et al., 2018; Gallant et al., 2020; Simon and Nader-Grosbois, 2021; Simon et al., 2025; Steinbeis, 2016). This finding is partly consistent with previous studies showing that children aged 3 to 7 could detect social exclusion and infer that excluded characters felt sadder than included characters, although they did not show clear emotional empathy for the excluded character (Song et al., 2015; Stengelin et al., 2022). In contrast, the present study found that 5-year-olds reported sadness in response to the excluded child’s experience, which is consistent with evidence from studies using more socially meaningful peer-exclusion scenarios (Masten et al., 2010). Evidence from older children also suggests that social appraisal processes shape emotional and bystander responses to exclusion, although such findings should not be generalized directly to preschoolers (Jones and Rutland, 2020). One possible explanation for differences across studies is variation in ecological validity. The two prior studies used moving waterdrop-like figures to simulate social exclusion (Song et al., 2015; Stengelin et al., 2022), which may have made it difficult for children to view the figures as intentional social agents with emotions and mental states. By contrast, the present study used a peer-interaction exclusion scenario that more closely resembled everyday play, which may have made the excluded child’s distress more salient and more likely to elicit emotional empathy.

Behavioral empathy did not differ significantly by age, and a larger proportion of 3- to 5-year-olds reported willingness to help than verbally demonstrated exclusion detection. This pattern suggests that helping intentions may not require fully explicit, verbally articulated exclusion detection. Younger children, especially 3-year-olds, may show implicit or partial sensitivity to exclusion when they notice that one player receives fewer turns or is left alone, which could lead them to help or include that player without explicitly stating that exclusion occurred (Marinović et al., 2017; Prendergast, 2019; Song et al., 2015). Task order may also have contributed. Children completed the exclusion detection task before the empathy and moral evaluation tasks, so questions about what happened, how the target child felt, and why may have directed attention to the excluded player and encouraged reflection. This interpretation is consistent with evidence that children’s responses to exclusion vary across detection, social evaluation, partner preference, and inclusion behavior tasks (Hwang and Markson, 2020; Stengelin et al., 2022; Woodward et al., 2022). Finally, behavioral empathy assessed helping intention or motivation, which may be more influenced by task prompts and familiar prosocial scripts than emotional empathy or cognitive empathy. When asked what they would do if they were next to Player 3, children may have inferred that Player 3 needed help and given a socially expected prosocial response. Because early helping can be elicited by concrete cues to need even when children’s explicit causal understanding is incomplete (Beier et al., 2019; Paulus, 2018; Smith-Flores et al., 2023), these findings should be interpreted cautiously. They may reflect early helping scripts and sensitivity to another child’s disadvantaged position rather than a fully explicit understanding of social exclusion.

The mediation findings suggest that moral evaluation is a candidate socio-cognitive mechanism linking exclusion detection to empathy (Rudert and Greifeneder, 2016; Rutland et al., 2024; Walker et al., 2022). Exclusion detection may allow children to notice that one player has been left out of the interaction, but this perceptual understanding alone may not be sufficient to elicit empathic responding. Children also need to evaluate the social meaning of the event, including whether the exclusion is unfair, harmful, or morally wrong. This interpretation is consistent with appraisal theories of empathy, which propose that vicarious emotional responses depend on how observers appraise the meaning and consequences of an event, as well as who is responsible for it (Wondra and Ellsworth, 2015). It is also consistent with developmental theories of emotion understanding, which view children’s understanding of others’ emotions as a third-person appraisal process involving interpretations of goals, outcomes, responsibility, and social norms (Doan et al., 2025). Thus, when children detect exclusion and evaluate it as morally wrong, they may be more likely to feel sad for the excluded child, understand the child’s emotional state, and report willingness to help.

These findings have both theoretical and practical implications. Theoretically, the present study extends research on preschoolers’ responses to social exclusion by integrating exclusion detection, moral evaluation, and three dimensions of empathy within a single developmental framework. The findings suggest that children’s empathy for an excluded peer is not simply a direct response to observing exclusion but is shaped by how they interpret the moral meaning of the event. Practically, the results suggest that early childhood educators may promote empathy for socially excluded peers by helping children identify exclusion in peer interactions, reflect on why exclusionary behavior is unfair or harmful, and consider how excluded children may feel (Doan et al., 2025; Killen et al., 2022; Simon and Nader-Grosbois, 2021; Soliman et al., 2021). Storybook discussions, role-play, and guided conversations about peer inclusion may be useful ways to scaffold children’s exclusion detection, moral evaluation, emotional empathy, cognitive empathy, and behavioral empathy (Ciesielska et al., 2025; Killen et al., 2022; Soliman et al., 2021).

Several limitations should also be noted. First, the cross-sectional design limits conclusions about developmental change and causal direction. Longitudinal research is needed to examine how exclusion detection, moral evaluation, and empathy influence one another over time. Second, empathy was assessed through children’s verbal responses and reported helping intentions; future studies should include behavioral observations and multi-informant assessments. This limitation is particularly important for behavioral empathy, because reported willingness to help may not correspond directly to actual helping or inclusion behavior. Third, the study focused on a Cyberball-based exclusion scenario, and future research should examine whether the findings generalize to more diverse real-world peer contexts, such as friendship groups, classroom play, and exclusion based on group membership. Fourth, although the two moral evaluation questions were counterbalanced, the broader task sequence was fixed. This fixed overall task order may have influenced later responses by directing children’s attention to the excluded peer before the empathy and moral evaluation questions. Future studies could counterbalance the broader task sequence or include control conditions to test this possibility. Finally, intervention studies are needed to test whether improving children’s exclusion detection and moral reasoning about exclusion can promote empathic concern and prosocial responses toward excluded peers.

## 5 Conclusion

Our study clarifies the development of social exclusion detection, moral evaluation, and empathy in preschool children. The results reveal age-related differences among children aged 3 to 5 years, with 5-year-olds showing stronger exclusion detection and cognitive empathy than 3-year-olds, and older preschoolers showing more negative moral evaluations of both the exclusionary behavior and the excluders. Children’s moral evaluations of exclusionary behavior partially mediated the link between exclusion detection and the three dimensions of empathy. In contrast, evaluations of the excluder mediated the relationship between exclusion detection and emotional and cognitive empathy, but not behavioral empathy. Overall, these results emphasize the important role of exclusion detection and moral evaluation in preschool children’s empathy toward socially excluded peers.

## Data Availability

The raw data supporting the conclusions of this article will be made available by the authors, without undue reservation.
